# Spatiotemporal Control of Intracellular Membrane Trafficking by Rho GTPases

**DOI:** 10.3390/cells8121478

**Published:** 2019-11-21

**Authors:** Monilola A. Olayioye, Bettina Noll, Angelika Hausser

**Affiliations:** Institute of Cell Biology and Immunology and Stuttgart Research Center Systems Biology, University of Stuttgart, Allmandring 31, 70569 Stuttgart, Germany; monilola.olayioye@izi.uni-stuttgart.de (M.A.O.); bettina.noll@izi.uni-stuttgart.de (B.N.)

**Keywords:** Rho GTPases, secretory pathway, endocytosis, cancer

## Abstract

As membrane-associated master regulators of cytoskeletal remodeling, Rho GTPases coordinate a wide range of biological processes such as cell adhesion, motility, and polarity. In the last years, Rho GTPases have also been recognized to control intracellular membrane sorting and trafficking steps directly; however, how Rho GTPase signaling is regulated at endomembranes is still poorly understood. In this review, we will specifically address the local Rho GTPase pools coordinating intracellular membrane trafficking with a focus on the endo- and exocytic pathways. We will further highlight the spatiotemporal molecular regulation of Rho signaling at endomembrane sites through Rho regulatory proteins, the GEFs and GAPs. Finally, we will discuss the contribution of dysregulated Rho signaling emanating from endomembranes to the development and progression of cancer.

## 1. Introduction

Intracellular membrane trafficking along endocytic and exocytic pathways is essential for the transport of proteins and membranes in all eukaryotic cells and contributes to the maintenance of cellular homeostasis. Transport carriers, such as vesicles and tubules that form and bud from donor membranes are then transported along microtubules and actin filaments and finally fuse with their target membrane. These sequentially occurring steps require a high degree of control at the molecular level. The small GTPases of the Rab and Arf families, which are known to control cargo sorting, vesicle fission, targeting, and fusion, are players that are traditionally investigated in this process (reviewed in [1]). In the last years, it has emerged that also the Rho GTPase family, a master regulator of cytoskeletal reorganization, plays an important role in membrane trafficking [2]. While it was initially assumed that this function is exclusively cytoskeleton-dependent, it is now evident that Rho GTPases regulate membrane trafficking at multiple levels through diverse downstream effector pathways. Considering that Rho signaling itself is initiated at cellular membranes, any perturbations of membrane trafficking and sorting caused by aberrant Rho GTPase signaling at endomembranes can potentially impact Rho GTPases at distal sites.

**Figure 1:**
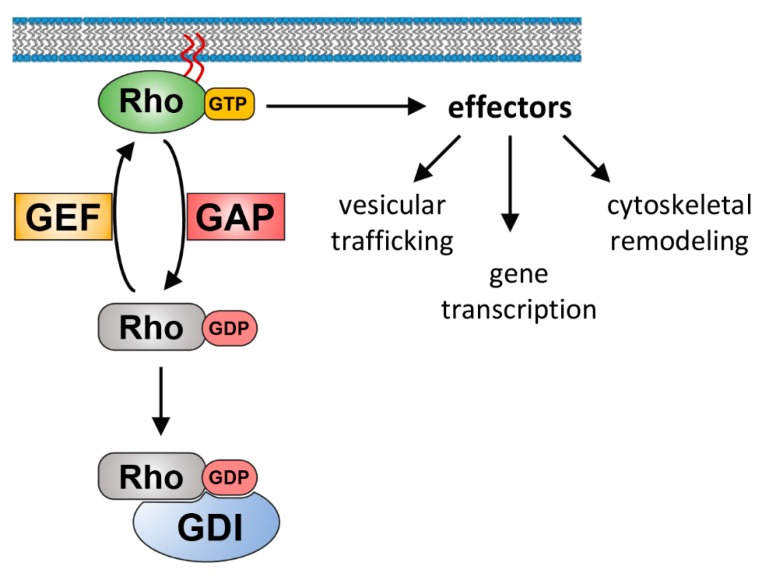
The classical Rho GTPase activity cycle and it’s regulation through GEFs, GAPs, and GDIs. Rho GDP/GTP cycling is tightly regulated by GEFs that promote the formation of the active GTP-bound form through exchanging GDP for GTP. On the contrary, GAPs catalyze the intrinsic GTPase activity and promote the formation of inactive GDP-bound Rho. In its inactive state, GDIs sequester Rho GTPases in the cytosol. Active Rho is associated with membranes through a hydrophobic lipid tail and, by binding to its effector proteins, controls several cellular processes.

The Rho GTPase family belongs to the Ras superfamily of small guanine nucleotide-binding proteins. In mammals, the Rho GTPases consist of 20 members that are, based on their structure, sequence, and function, further divided into eight subfamilies (Rho, Rac, Cdc42, Rnd, RhoD/F, RhoU/V, RhoH, and RhoBTB) (reviewed in [3,4]). The best-characterized family members up to date are RhoA, Rac1, and Cdc42, and much of our current understanding of the biochemistry and biology of the Rho family has come from the extensive investigation of these proteins. Rho, Rac, and Cdc42 family members belong to the classical Rho GTPases, which act as bi-molecular switches by cycling between an inactive GDP-bound and an active GTP-bound state. Rho GDP/GTP cycling is tightly regulated by guanine nucleotide exchange factors (GEFs) that promote the formation of the active GTP-bound form through exchanging GDP for GTP. On the contrary, GTPase-activating proteins (GAPs) catalyze the intrinsic GTPase activity and promote the formation of inactive GDP-bound Rho. This inactive state can be subsequently recognized by guanine nucleotide dissociation inhibitors (GDIs), which sequester Rho GTPases in the cytosol [5,6] (Figure 1). Whereas classical Rho GTPases are regulated by GDP/GTP cycling, atypical Rho GTPases are regulated by other mechanisms that occur in particular at the transcriptional and post-translational level [7]. In the case of atypical Rho GTPases, GTP is usually constitutively bound, either because these Rho GTPases possess high intrinsic nucleotide exchange activity or have substitutions in their GTPase domain that prevent GTP hydrolysis.

In addition to their core guanine nucleotide-binding domain, most of the Rho GTPase family members contain a hypervariable region and a C-terminal *CAAX* tetrapeptide motif (where C represents cysteine, A is an aliphatic amino acid, and *X* is any amino acid), which triggers prenylation. This hydrophobic lipid tail promotes proper subcellular localization of active Rho GTPases to the plasma membrane (PM) and endomembranes [8]. The intracellular distribution of some Rho GTPases such as RhoB, TC10 (RhoQ), and TCL (RhoJ) is further controlled by the dynamic regulation of membrane association through the addition of palmitoyl modifications [9]. A subset of Rho GTPases additionally possess a polybasic sequence in their hypervariable region supporting the interaction with negatively charged membranes [10,11]. Polybasic sequences are thought to be relatively unselective in terms of the lipid composition of the membrane they bind to, with interactions mainly mediated by charge differences. Accordingly, differences in net positive charge of the hypervariable regions of Rac1 and Rac2 GTPases were shown to account for the recruitment to the highly negatively charged PM and intermediately charged phagosome membrane in primary neutrophils, respectively [12]. More recent studies using super-resolution microscopy and manipulation of PM lipid composition demonstrated that the localization of the polybasic prenylated membrane anchoring sequence of Rac1 depended on the lipid messengers phosphatidylinositol 3,4,5-trisphosphate and phosphatidic acid. This suggests a certain degree of lipid recognition that goes beyond simple electrostatic interactions [13,14]. Finally, phosphorylation, transglutamination, and AMPylation modulate Rho GTPase signaling by altering Rho GTPase localization, activity, and interaction with protein partners while ubiquitinylation regulates protein stability and turnover (reviewed in [7,15,16]).

Here, we will focus on how local Rho GTPase pools are implicated in membrane trafficking, along the endo- and exocytic pathways. We will further highlight the spatiotemporal molecular regulation of Rho signaling at these endomembranes through GEFs and GAPs. Finally, we will discuss how dysregulated Rho signaling emanating from endomembranes contributes to the development and progression of cancer.

## 2. Spatial Organization of Rho GTPase Signaling Complexes

Association of Rho GTPases to cellular membranes restricts their activities to specific locations in the cells. Although membrane localization of Rho GTPases is primarily determined by intrinsic sequences present in the Rho GTPases and the combined effects of post-translational lipid modifications, these features do not always sufficiently explain how Rho GTPases are targeted to specific subcellular sites and how localized Rho GTPase activity gradients are achieved. Scaffolding proteins and Rho regulatory proteins, which also display distinct intracellular distributions, additionally recruit and contribute to the spatial organization of Rho GTPase signaling complexes. For example, at the leading edge of the PM, active Rac1 nanoclusters are formed by associating with the RacGEF T-cell lymphoma invasion and metastasis-inducing protein 1 (Tiam1) and the Rac1 effector WASP verprolin homology protein 2 (WAVE2) [17], whereas Cdc42 is known to localize to Golgi membranes via binding the vesicle-coat protein coatomer [18,19,20]. In fact, besides mediating membrane binding, the hypervariable region also engages in various protein interactions that control both the targeting and signaling specificity of Rho GTPases, as has been reviewed in detail for Rac1 [21]. Finally, RhoGDIs are also not only passive shuttles that keep inactive Rho GTPases in the cytoplasm, but they rather actively contribute to the spatiotemporal patterning of Rho GTPases by removing active Rho GTPases from the PM [22].

Active Rho GTPases initiate signaling cascades by binding to downstream effectors. To date, more than 60 effector proteins of Rho GTPases have been identified, and their specific expression profile determines the cellular response towards Rho activation [3,23]. The most prominent function of Rho GTPases is the regulation of actin polymerization through effector proteins such as actin nucleators of the formin and Wiskott-Aldrich syndrome protein (WASP) family, as well as p21-activated kinase (PAK) and Rho-associated protein kinase (ROCK) kinases (for a review see [3,24]). Additionally, the organization and dynamics of microtubuli and vimentin intermediate filaments, as well as the crosstalk between these cytoskeletal elements and the actin cytoskeleton is regulated by Rho GTPases [25,26,27]. Rho GTPases also interact with various kinases and phospholipases, e.g., protein kinase N (PKN) and phospholipase C epsilon (PLCε), thereby directly and indirectly affecting the lipid composition of membranes [28,29,30,31]. Through this diverse network of effector proteins, Rho GTPases contribute to membrane trafficking and sorting at multiple levels, e.g., by promoting actin polymerization on endomembranes by driving the movement of membrane carriers along microtubules, or by changing the local lipid composition to facilitate vesicle budding. Indeed, in addition to their prominent localization at the PM, distinct Rho GTPases, GEFs, GAPs, and Rho effectors have been detected along with the endo- and exocytic pathways. However, the precise spatiotemporal regulation of these subcellular Rho signaling complexes is only poorly understood. The association of Rho GTPases with endo- and exocytic carriers can be interpreted as a way of delivering inactive Rho GTPases to or removing active Rho GTPases from their main site of action, the PM. Indeed, Rho GTPases are trafficked themselves, as shown for RhoB, which internalizes from the PM and is shuttled to the endolysosomal compartment for degradation or is recycled back to the PM to drive amoeboid migration [32]. Tracking of a photoactivatable RhoB variant specifically activated at the PM provided evidence for an endosomal RhoB pool originating from the PM. FRAP experiments further revealed low recovery of fluorescence on vesicles after photobleaching, suggesting little exchange of internalized RhoB between vesicles and the cytoplasm [32]. However, there is also mounting evidence for the coexistence of subcellular Rho pools that are regulated independently of one another, including activation and inactivation cycles occurring directly at endomembrane sites. For example, in the case of Cdc42, it has been proposed that Cdc42-GTP generated at the Golgi membranes is independently regulated from the PM pool by a Golgi-localized signaling network, but can then provide a reservoir for Cdc42-GTP required at the PM [33]. Given the interdependence of Rho GTPase signaling and trafficking, experimental investigations on how distinct Rho GTPase pools generate localized signaling outputs and how these are connected with biological responses remain a major challenge.

## 3. Spatiotemporal Rho Regulation by GEFs and GAPs

GEFs and GAPs are signaling nodes that receive multiple input signals to modulate the amplitude and duration of Rho GTPase activity and trigger appropriate downstream responses. The fact that the number of GEFs, GAPs, and GDIs greatly exceeds the number of Rho GTPases can be explained by the required control of Rho activation in space and time, preventing inappropriate or prolonged signaling [34,35]. In terms of domain organization, RhoGEFs contain conserved tandem Dbl homology (DH)–pleckstrin homology (PH), or dedicator of cytokinesis (DOCK) domains. GEFs containing a tandem DH-PH domain confer nucleotide exchange activity towards Rho proteins via the DH domain and bind to membranes with a differential affinity for phosphoinositide species through the PH domain [36,37]. RhoGAP proteins also commonly include lipid-binding domains such as PH, C2, phox homology (PX), or Bin1/amphiphysin/RVS167 (BAR) domains in addition to their conserved RhoGAP domain [37]. It is still poorly understood how the specificity of GEFs and GAPs for certain Rho GTPases is achieved, which is, in some cases, very broad, whereas other Rho regulators control only one specific GTPase [6]. An additional layer of complexity in Rho regulation is provided by the fact that GEFs and GAPs are also regulated by posttranslational modifications and protein interactions, whereby localization, specificity, and activity are modulated [6,7]. The large variety, subcellular distribution, and ability of Rho regulators to form protein complexes, together with the GTP-GDP cycling and posttranslational regulation of Rho GTPases creates a complex network of interactions determining the precise spatiotemporal activation of Rho GTPases and as a consequence, the cellular outcome [7].

The RhoGAP protein Deleted in liver cancer 3 (DLC3, STARD8) is an excellent example for such complex regulation of Rho GTPase signaling. In polarized epithelial cells, DLC3 is targeted to adherens junctions by the scaffold protein Scribble to suppress RhoA signaling [38]. By additionally recruiting the RacGEF β-Pix (ARHGEF7), Scribble contributes to the establishment of antagonizing RhoA and Rac activity gradients along the apical-basal axis of epithelial cells [39,40]. By contrast, in motile epithelial cells, DLC3 switches adaptors and predominantly localizes to endomembranes through interactions with sorting nexin 27 (SNX27). Here, DLC3 was found to regulate RhoB signaling [41,42]. Another example of localized Rho regulation is GEF-H1 (ARHGEF2), a microtubule-associated RhoGEF that when released from microtubules, stimulates the activation of RhoA and RhoB [43,44]. In HeLa cells, GEF-H1 interacts with the exocyst component Sec5 leading to local RhoA activation at the PM, thereby promoting exocyst assembly and exocytosis [45]. Apart from this, GEF-H1 also promotes the fission of carriers at the trans-Golgi network [46]. In motile cells, GEF-H1 activity is controlled by phosphorylation through the protein tyrosine kinase c-Src, and this is critical for RhoA activation at the leading edge [47]. However, GEF-H1 is also implicated in the endosomal trafficking of c-Src [44]. Furthermore, IQGAP1, a large scaffold protein and master regulator of several small GTPases, has been reported to recruit Rho regulators, like Tiam1 and RacGAP1, and Rho effector proteins, like N-WASP, Arp2/3, and Dia, to modulate spatial Rho signaling at the leading edge [48].

In excitatory glutamatergic hippocampal neurons, the synaptic RhoGEF Kalirin (KALRN) interacts with the core-scaffold machinery of the postsynaptic density (PSD) and clusters proteins essential for GTP signaling to regulate the activity of glutamate receptors [49]. Finally, Tiam1 and the RacGAP Bcr form a complex within the PSD and act in concert to coordinate Rac1-mediated receptor endocytosis during excitatory synapse development [50].

Consequently, it is not surprising that dysregulated expression or altered activity of Rho regulators caused by epigenetic, somatic mutations, or posttranslational modifications are associated with disease and can contribute to the neoplastic transformation of cells [51].

## 4. Rho GTPases Acting along the Secretory Pathway

Most of the cellular transmembrane proteins and secreted soluble proteins traffic through the secretory pathway. These proteins start their journey in the endoplasmic reticulum (ER) and are transported via the ER-to-Golgi-intermediate compartment (ERGIC) to the Golgi complex. Vesicular carriers are then transported from the trans-Golgi network or endosomal compartments to specific sites of the PM. This timely, organized multistep process is accomplished through the coordinated actions of the membrane trafficking machinery and the cytoskeleton orchestrated by a plethora of signaling molecules (reviewed in [52]).

Several studies using diverse methods, including fractionation, immunofluorescence, and electron microscopy, have identified Cdc42 as the first Rho GTPase found to localize to the Golgi complex [18,53,54,55]. For a long time, Cdc42 was thought to be the only Rho GTPase with a specific function at this compartment.

Several reports demonstrated that Cdc42 is important for intra-Golgi transport [53,56,57,58]. Specifically, Cdc42 interacts with the coat protomer I (COPI)–vesicle coat protein, coatomer [19,20], which is responsible for promoting retrograde transport from the Golgi to the ER, however, Cdc42 also binds to cargo proteins transported in both the anterograde and retrograde directions [58]. In response to extracellular cues, active Cdc42 promotes anterograde and inhibits retrograde intra-Golgi transport. This can be explained by active Cdc42 competing with COPI for binding to retrograde but not anterograde cargoes [58]. Although the signaling pathways directing Cdc42 activation in intra-Golgi transport remain mostly elusive, the Cdc42/COPI interplay provides an example of how cells synchronize their secretory trafficking capacity with extracellular demands. Moreover, in polarized epithelial cells, Golgi-localized Cdc42 was shown to regulate polarized secretion in an actin cytoskeleton-dependent manner. Here, Cdc42 mutants affected vesicle formation at the trans-Golgi network (TGN), stimulating the release of apical cargo and inhibiting the release of basolateral cargo [59].

The presence of active Cdc42 at the Golgi complex was indirectly proven by the visualization of the Cdc42 effector proteins Arp2/3 and N-WASP at this compartment [53,60]. Importantly, live-cell imaging using biosensors has provided spatial information on the activity of Cdc42 at the Golgi complex [33,61]. The development of a new generation of genetically encoded fluorescence resonance energy transfer (FRET) biosensors with high selectivity, sensitivity, and dynamic range has further pushed the field forward [62,63,64].

While the presence of active Cdc42 at Golgi membranes has been demonstrated, the identification of the GEFs and GAPs responsible for regulating Cdc42 activity at this compartment still remains a challenge. The only Cdc42 GAP identified at the Golgi so far is ARHGAP21 (also known as ARHGAP10), which is recruited to Golgi membranes via active Arf1. At the Golgi, ARHGAP21 regulates actin polymerization through the Cdc42 effector N-WASP and the Arp2/3 complex [60]. Additionally, the FMNL2/3 formins were identified as effector proteins of Cdc42 at Golgi membranes [65]. While it is clear that actin polymerization is required for Cdc42 controlled intra-Golgi transport, it is not understood how these different actin assembly pathways are connected to coordinate anterograde trafficking downstream of Cdc42.

Up to date, four Cdc42-specific GEFs, Tuba (DNMBP), faciogenital dysplasia 1 protein (FGD1), intersectin-1 (ITSN1), and Dbs-130 (MCF2L) have been reported to localize to the Golgi. Endogenous Tuba was initially observed at the Golgi membranes in rat brain cryosections and in HeLa cells using indirect immunofluorescence and co-staining with GM130 [66,67] and has been reported to regulate Cdc42 activity in this compartment [62]. By performing fluorescence lifetime-resolved imaging microscopy (FLIM)-FRET measurements in living cells, Sütterlin and co-workers showed that the activity of Golgi-localized Cdc42 was regulated by ARHGAP21 and Tuba but not FGD1 [62].

FGD1 is a selective Cdc42 GEF, and mutations in the FGD1 gene are responsible for the X-linked disorder known as faciogenital dysplasia. GFP-tagged FGD1 is located at the trans-Golgi network in HeLa and COS-7 cells, as well as in MC3T3 osteoblasts, and its loss impairs the formation of post-Golgi carriers in a Cdc42 dependent manner [56,68,69]. A very recent study aimed to identify the Golgi-localized Cdc42 effectors involved in FGD1-mediated post-Golgi transport. However, overexpression of effector-specific Cdc42 mutants with specific activities for PAK1, Ras GTPase-activating-like protein IQGAP1, N-WASP, or partitioning defective 6 homolog (PAR6) only partially rescued membrane trafficking in FGD1-deficient cells, suggesting that the integrated activities of several downstream targets of Cdc42 are required to support FGD1-mediated export from the Golgi [70]. In HeLa cells, endogenous Dbs-130 has been detected at the Golgi complex, where its inhibition limited Cdc42 activity and was associated with an enlarged Golgi [71]. Whereas normal protein transport from the ER to the Golgi was not affected by Dbs-130 inhibition, transport from the Golgi to the PM was disturbed [71]. While the GFP-tagged Sec14 domain of Dbs-130 convincingly localized to the Golgi complex, the specificity of the antibody used to detect endogenous Dbs-130 in cells has not sufficiently been proven. In this context, a very recent report provided convincing evidence that in Hela cells, endogenous intersectin-1 is recruited to the Golgi complex through interaction with GRIP and coiled-coil domain containing 88 kDa (GCC88), a TGN-located tether protein, and this increased the F-actin network and actin-myosin forces at the TGN [72]. Notably, a small molecule that targets the Cdc42-intersectin-1 interaction disrupted Golgi membranes [73]. Together, these data make intersectin-1 an attractive candidate for coordinating Cdc42 activity at the Golgi complex.

Despite these reports, the localization of RhoGEFs at Golgi membranes is still a matter of debate. In many reports, endogenous and overexpressed Tuba failed to be detected at the Golgi but rather was present on vesicle-like structures [33,74,75,76] questioning the specificity of the antibodies used in the former studies. Additionally, a recent study confirmed a perinuclear/vesicular localization for overexpressed intersectin-1, while FGD1 was mainly cytosolic in primary endothelial cells, although it was not investigated whether the RhoGEFs activate Cdc42 at these sites [76]. Finally, Rocks and colleagues comprehensively characterized in a new study the RhoGEFs and RhoGAPs, including their substrate specificities, and subcellular localization. Surprisingly, ‘non-canonical’ locations, comprising the Golgi, lysosomes, endomembranes, and the endoplasmic reticulum, were decorated by RhoGAPs, but no evidence for Rho activation at these structures was found [77]. However, the overexpression of GEFs (and GAPs) used in this study might distort the localization of the endogenous proteins. Conversely, the use of antibodies for detecting endogenous proteins by indirect immunofluorescence analysis requires high-quality standards, including testing for specificity by performing knockdown experiments to exclude artifacts, which is, in most cases, not presented.

The above described conflicting results on the localization of Cdc42 GEFs could suggest that the regulation of Cdc42 activity at the Golgi is highly cell type-specific. Alternatively, the presence of only small pools of GEFs could be transient in nature, and thus, difficult to detect using fluorescence analysis of fixed cells. Here, the development of novel fluorescent biosensors that report GEF activity in combination with quantitative analysis of live-cell imaging data will reveal the relationship between local GEF and RhoGTPase activities [47].

Of note, coronin7 and GM130, two resident Golgi proteins without typical GEF and GAP domains, were proposed to contribute to Cdc42 regulation at the Golgi [66,78]. Coronin7 is implicated in vesicle formation and cargo export from the Golgi [79] and seems to limit steady-state Cdc42 activity to maintain Golgi integrity [78]. GM130 binds and sequesters RasGRF2, a RasGEF that inhibits Cdc42 activity, thereby contributing to Cdc42 activation [33]. Loss of GM130 did not affect ER-Golgi trafficking, Golgi-to-PM trafficking, or coatomer recruitment to the Golgi but reduced the presence of active Cdc42 at the leading edge of migration cells [33]. It, thus, seems that Cdc42 activated at the Golgi membranes downstream of GM130 replenishes the PM pool of active Cdc42 required for directional cell migration and maintenance of cell polarity. Indeed, in fission yeast, active Cdc42 is present on secretory vesicles and delivered to sites of polarized growth [80]. Likewise, in mammalian cells, Cdc42 has been detected on Arf6-positive intracellular vesicles, which are trafficked en route to the leading edge of migration cells [55]. Notably, a connected Golgi ribbon was necessary for Cdc42 activity at the PM, further confirming the postulated role of the Golgi-localized Cdc42 in replenishing PM Cdc42 pools [62].

In addition to Cdc42, other members of the Rho GTPase family have been reported to localize to the Golgi complex. Early studies on GFP-tagged RhoA showed a strong cytoplasmic expression with some enhancement in the Golgi region [81], suggesting that only a low amount of RhoA is present at Golgi membranes. On the contrary, RhoB was localized in a discrete perinuclear compartment that colocalized with Golgi markers [81]. Under steady-state conditions, the activity of RhoA at the Golgi is low [82]. This can be considered as a safeguard mechanism because the hyperactivation of RhoA has been linked to Golgi complex fragmentation [83,84]. Upon nocodazole and lysophosphatidic acid (LPA) treatment, active RhoA was detected at Golgi membranes in HeLa cells and at Golgi outposts in hippocampal neurons, respectively, by using a FRET-based RhoA biosensor in combination with a Golgi marker [46,85]. So far, the only RhoA-specific GAP protein detected at Golgi membranes is DLC3 [41]. The depletion of DLC3 in HeLa cells strongly increased RhoA activity at Golgi membranes and led to a fragmented phenotype underscoring the importance of tightly controlled local RhoA activity [41]. We recently showed that activation of protease-activated receptors at the PM triggered RhoA activity at the Golgi in a GEF-H1 dependent manner [46]. However, as GEF-H1 is localized to microtubules [86] and could not be convincingly detected on Golgi membranes, the question of how local RhoA activation is achieved is still open. We further demonstrated that Golgi-localized RhoA activity was associated with protein kinase D (PKD)-dependent formation of post-Golgi carriers that delivered cargo to focal adhesions [46]. Also RhoB was able to activate PKD at this site [46]; however, whether it also has a role in exocytosis still needs to be investigated.

In hippocampal neurons, Golgi-localized RhoA was shown to be activated downstream of LPA, a bioactive lipid that signals through defined G-Protein coupled receptors (GPCRs) [85]. In this study, the nature of the GEFs and GAPs responsible for RhoA activation remained elusive; however, downstream activation of ROCK and PKD1 was demonstrated to be required for the polarized formation of Golgi outposts [85]. In this respect, a promising candidate RhoGEF is the triple functional domain protein (TRIO), which has specificity for RhoA, Cdc42, and Rac1. Very recently, TRIO has been detected at the Golgi complex of neuronal cells where it is involved in membrane trafficking and neurite outgrowth [87], functions for which Golgi outposts are essential [88]. In contrast, in HeLa cells, ROCK1 and 2 were dispensable for RhoA-mediated PKD activation; instead, the phospholipase PLCε was identified as the key Rho effector in this regard [46]. By activating PLCε, TGN membranes become enriched in diacylglycerol, which in turn recruits and activates a master regulator of vesicle fission at the TGN, PKD [46]. RhoA, thus directly affects the lipid composition of the TGN membranes, thereby facilitating the assembly of the molecular trafficking machinery. At present, the reason(s) underlying the differential regulation of PKD downstream of RhoA in HeLa cells and neurons is not clear. In this respect, it is worth mentioning that the brain-specific Rho binding protein Citron-N is localized to the Golgi where it controls actin dynamics by assembling ROCK and the actin-binding, neuron-specific protein Profilin-IIa [89,90]. This highlights that the presence of the respective GEFs, GAPs, and effector proteins dictates the signaling pathways initiated by Rho GTPase activity at Golgi membranes. 

RhoBTB3 represents an atypical Golgi-localized Rho GTPase. Pioneer work by Suzanne Pfeffer’s lab showed that unlike most Rho-related GTPases, RhoBTB3 binds and hydrolyzes ATP instead of GTP [91]. At the TGN, RhoBTB3 acts as a Rab9 effector protein and is required for the transport of mannose-6-phosphate-receptors from late endosomes back to the Golgi, suggesting a role for the ATPase in vesicle docking events [91]. An unexpected finding was that RhoBTB3 binds cyclin E at Golgi membranes, presenting it to a Golgi-localized cullin-3–ubiquitin ligase complex, whereby cyclin E ubiquitylation and S/G2 transition of the cell cycle were promoted. Depletion of RhoBTB3 arrested cells in the S phase, triggered Golgi fragmentation, and elevated cyclin E levels [92]. Golgi membranes might thus serve as a platform that integrate organelle integrity and secretory function with cell cycle control.

Another Rho GTPase that has been located at trans-Golgi membranes is the atypical member RhoE/Rnd3. RhoE inhibited RhoA signaling in part by binding to ROCK1, thereby preventing it from phosphorylating its targets. In turn, RhoE activity is itself regulated by phosphorylation by ROCK I on multiple sites [93,94].

Last but not least, trans-Golgi-localization of the atypical family member RhoD has been reported as well. RhoD cycles between active and inactive conformations in the absence of GEFs and GAP [95,96]. It functions through interaction with WASP homolog-associated protein with actin, membranes, and microtubules (WHAMM), and both are required for efficient Golgi to PM transport of cargo proteins [95,96]. WHAMM stimulates Arp2/3-mediated actin polymerization both at the Golgi apparatus and along tubular membranes, and its activity in membrane tubulation requires F-actin and interaction with microtubules [96]. Notably, Rab1 is required for WHAMM localization to the Golgi and tubular membranes; however, Rab1 inhibits WHAMM-mediated actin assembly [97], possibly by competing with RhoD binding.

So far, only a few RhoGTPases have been associated with exocytic vesicles. Cdc42 has been detected on Arf6-positive vesicles trafficking to the leading edge of migrating cells, thereby contributing to the maintenance of cell polarity [55]. The Cdc42 subfamily member TC10 plays a significant role in the exocytosis of GLUT4 [98] and other proteins [99,100] through its effector proteins Exo70, a component of the exocyst complex [101] and the protein interacting specifically with Tc10 (PIST; also known as GOPC), a Golgi localized receptor-targeting protein [100]. Early reports showed localization of TC10 to vesicular structures [81], which were later identified as exocytic vesicles [102,103]. GTP hydrolysis by TC10 was required for the fusion of these vesicles with the PM. Specifically, the vesicle tethering occurred through Exo70 induced assembly of the exocyst complex. Subsequently, on stimulation, p190RhoGAP (ARHGAP35) accelerated GTP hydrolysis of TC10 on these vesicles, inducing the release of Exo70 and exocyst disassembly, thereby promoting vesicle fusion. TC10 also localized to a perinuclear compartment, presumably the Golgi complex, and it is supposed that through its interaction with PIST, it could play a role in cargo loading at this compartment [102]. The GEF activating TC10 has not been identified so far, but in this respect, it is interesting that PIST is an effector protein for Rab6 [69], a Golgi-localized Rab being also present on exocytic vesicles [104]. Notably, the RhoGEF ARHGEF10 has been detected on Rab6-positive vesicles [105,106], representing, thus an attractive candidate for TC10 activation on these structures.

## 5. Rho GTPases Acting along the Endocytic Pathway

The endocytic pathway starts with the internalization of macromolecules and surface proteins at the PM. Once intracellular vesicles have formed, internalized cargo is transported and sorted via a pleiomorphic series of tubulovesicular and interconnected compartments, including early, late, sorting, and recycling endosomes as well as lysosomes. By this means, cargo can be stored, recycled back to the PM, or routed to the endolysosomal system for targeted degradation. It is now accepted that specific endomembranes form signaling platforms that dynamically and efficiently translate extracellular signals into biological outcomes. Whereas the general function of Rho GTPases in internalization has been intensively characterized and reviewed in detail elsewhere [2,107,108,109], much less is known about the specific Rho GTPases and respective GEFs and GAPs involved in local Rho regulation along the endocytic pathway. In this part, we will thus highlight emerging research about Rho GTPase signaling and regulation at endosomal membranes and how this impacts endosomal dynamics, cargo sorting, cell signaling, and behavior.

Among the Rho GTPases, RhoB was the first member found to localize to endosomes [31,110]. Pioneering work over the last two decades further demonstrated a reliable role for RhoB in the regulation of endosomal trafficking mainly through actin cytoskeleton remodeling. In detail, Gampel and colleagues showed that constitutive active endosomal RhoB delayed the trafficking of the epidermal growth factor receptor (EGFR) to late endosomes [31], and that internalized EGFR activated endogenous RhoB once arriving at RhoB-positive endosomes through the actions of the GEF Vav2 [111]. The Ser/Thr kinase C-related protein kinase (PRK1) was then identified to be recruited to endosomes by RhoB, where its activation impacted trafficking, degradation, and signaling of the EGFR; however, actin dependency was not investigated in this context [30]. Additionally, a function of RhoB in regulating degradation and signal termination of the chemokine receptor CXCR2 was demonstrated suggesting a more general role for RhoB in receptor trafficking [112]. Mechanistically, actin nucleator Diaphanous-related formin 1 (Dia1) was later shown to mediate actin polymerization downstream of endosomal RhoB [113]. Last but not least, mDia2 was also shown to associate and interact with RhoB on endosomes, where it controlled actin dynamics and vesicular trafficking [114]. Along these lines, a RhoB-dependent mechanism of movement and activation of the cytoplasmic tyrosine kinase Src “en route” to the PM was described, which was also dependent on Scar1 (WAVE1)-mediated endosomal actin polymerization. Knocking out RhoB suppressed both the catalytic activation of Src, as well as the translocation of the active kinase to peripheral membrane structures [115]. Of note, Dia proteins bind to Src via SH3 domains, potentially bringing Src together with its substrates, thereby affecting actin recruitment and endosomal dynamics [116]. Actin polymerization on endosomes might thus be initiated by Dia formins in a Src-dependent manner.

The type of prenylation of RhoB is thought to be one of the determinants targeting RhoB to endosomes. RhoB is unique within the Rho subfamily as the isoprenoid modification can be either a farnesyl group (like Ras) or a geranylgeranyl group (like other Rho proteins). Whereas the geranylgeranylated form of RhoB was predominately localized to late endosomes, the farnesylated form was detected mostly at the PM [117]. It is, thus tempting to speculate that endosome-specific RhoB represents a functionally distinct subset from the one existing at the PM. Of note, the identity, localization, and regulation of the prenylation factors responsible for differential RhoB prenylation are still elusive.

With respect to endosomal RhoB regulation, work from our lab has identified the Rho GAP protein DLC3 to localize to the Rab8-positive endocytic recycling compartment and to RhoB-positive endosomes [41,42]. By locally regulating perinuclear RhoA and endosomal RhoB activity, DLC3 maintains organelle integrity and regulates membrane transport. Knockdown of DLC3 impaired transferrin and EGF receptor endosomal trafficking, and this effect was restored by the co-depletion of RhoA and RhoB [41]. Here, depletion of DLC3 in HeLa cells enhanced perinuclear RhoA activity as assessed using a RhoA biosensor and caused vesiculation of the Rab8 recycling compartment and Golgi fragmentation. In TGFβ-treated MCF10A cells, we further observed early endosomal localization of DLC3. Using a genetically encoded FRET-based RhoB biosensor, we have provided evidence for the regulation of RhoB by DLC3 at vesicular structures [42]. The importance of RhoB regulation by DLC3 for endosomal trafficking was underscored by the excessive accumulation of F-actin at and trapping of the cargo protein MT1-MMP in early endosomal structures in cells depleted of DLC3 which was associated with aberrant Rab4-dependent MT1-MMP recycling and enhanced invadopodia matrix degradation [42].

RhoD is also implicated in actin-based endocytic vesicle movement. Murphy and colleagues demonstrated that the intracellular motility of endosomes was inhibited in cells overexpressing wild-type or active RhoD [118]. This was then linked to an actin-based and c-Src-dependent mechanisms because hDia2C, a splice variant of human Dia, was specifically bound and recruited to early endosomes by RhoD where it locally activated Src-kinase activity. By this, early endosomes were aligned along actin filaments, and their motility was reduced [119]. Given that RhoD until today has not been involved in the degradative pathway as it is the case for RhoB, RhoD might target a different subset of endosomes. This is in line with findings of Sandilands and colleagues, showing that the membrane targeting and spatial activation of Src kinase family members are influenced by palmitoylation, which determines their localization to RhoB and RhoD-positive endosomes, respectively [120]. However, as RhoD has also been shown to localize to Rab5-positive early endosomes [121] where it binds the Rab5 effector Rabankyrin-5 and regulates together with Rab5 receptor tyrosine kinase (RTK) trafficking [122], it is tempting to speculate that RhoB and RhoD might also control the same subset of endosomes by affecting each other. This could be reminiscent of the crosstalk between RhoB and Rac1 [123] or RhoB and Cdc42 [124]. Nevertheless, this potential relationship needs further investigation and might depend on the cellular context.

Rac1, one of the best-characterized Rho family members, was detected by several groups on endocytic vesicles in different eukaryotic organisms [125,126,127]. Studies on Rac1 activation and trafficking over the last years revealed an important role of the endocytic system in regulating spatiotemporal Rac1 functionality, some of which are summarized in the following. After growth factor-induced activation of mitogenic receptors such as c-MET, the receptor for a hepatocyte growth factor (HGF), Palamidessi and colleagues convincingly showed that clathrin-mediated endocytosis and Rab5 activity are required for Rac1 activation on early endosomes through Tiam1. Moreover, the recycling of active endosomal Rac1 back to the PM, via the small GTPase Arf6, triggered the formation of actin-based migratory protrusions. This endocytic trafficking route of active Rac1 through Rab5- and Arf6- positive compartments was linked to cell motility in a variety of tumor cells [127]. In line with this, it has been described that active Arf6 induces Rac1 activation through endosomal trafficking [128]. In addition, HGF stimulation also induced c-Met-dependent Rac1 activation in perinuclear, Rab7-positive endosomes by engaging the specific effectors PI3K and the GEF Vav2 to activate cell migration and invasion [125]. Additionally, Rab7 was shown to directly interact with Rac1 on endosomes, thereby enabling Rac1 activation and promoting Rac1 delivery to the PM to stimulate cell migration [129]. On the other side, RhoB was shown to act as a negative regulator of Rac1 activity. Inhibition of RhoB induced Rac1 activity, and consequently, lamellipodia protrusion [130]. This is in line with active RhoB retaining Rac1 in intracellular endosomal localization and preventing Rac1 activation and recycling to the cell border, whereby Rac1-dependent endothelial barrier reformation and stabilization of cell-cell junctions were blocked [123]. Another negative Rac1 regulator is the Rab11 effector FIP3, which was shown to recruit Rac1 to Rab11-positive recycling endosomes, thereby restricting access of Rac1 to the PM. In fact, FIP3 silencing induced T-cell spreading, a process that is controlled by Rac1, suggesting endosomal trafficking of Rac1 to regulate T-cell spreading and activation in the immunological synapse [131]. Finally, Rab8, which localizes to the endocytic recycling compartment, was demonstrated to increase Rac1 activity and Tiam1/Rac1 mobilization from intracellular compartments to cortical locations to maintain directionality of migrating cells by enabling focal adhesion turnover and actin polymerization [132]. Combined, these studies suggest that endosomal trafficking routes are major determinants of Rac activity patterns emanating from the PM.

Beyond being an important regulator of vesicle trafficking in the secretory pathway, Cdc42 is involved in retrograde Golgi-to-ER transport in an N-WASP-dependent manner as overexpression and activation of Cdc42 inhibited the retrograde transport of Shiga toxin from the Golgi apparatus to the ER [53]. In addition, activation of Cdc42 or knockdown of ARHGAP21 suppressed the retrograde transport of Shiga toxin from the cell periphery to the juxtanuclear Golgi region [57]. Most importantly, secramine was identified as a small molecule inhibitor of Golgi-to-ER transport, which seemed to act by inhibiting the Cdc42 association with membranes in a RhoGDI-dependent manner [133]. An analogous mechanism to the Rab5-Rac1-Arf6 endocytic signaling-axis was described for Cdc42 at the leading edge of astrocytes [55]. Here, localization of Cdc42 and its GEF β-PIX to early endosomes depended on Rab5, and the directed delivery of Cdc42 to the leading edge depended on Arf6, reminiscent of the scenario described for Rac1 [55,127].

Other less described Rho GTPases were found to reside at endosomes and to control the fate of membrane receptors. The Rho GTPase TCL was shown to be required for transferrin receptor recycling to the PM [134], and RhoG regulates lysosomal dynamics [135].

Last but not least, atypical Rho GTPases of the RhoBTB subfamily are also known to play a role in vesicular trafficking and are associated with endocytic membranes [136,137]. They are present mostly in an active, GTP-bound state at membranes, do not undergo GEF and GAP-dependent GTP-GDP cycling but are rather positively and negatively regulated at the transcriptional level, by protein-interactions and degradation [136,138]. RhoBTB3, for example, is involved in retrograde transport from endosomes to the Golgi apparatus by interacting with active Rab9 and TIP47 [91]. As RhoBTB3 also binds to an early endosomal protein that controls endosome-to-lysosome trafficking (hepatocyte growth factor-regulated tyrosine kinase substrate), it is speculated that it might participate in the sorting of membrane cargo proteins to multivesicular bodies for subsequent degradation in the lysosome [139].

**Figure 2:**
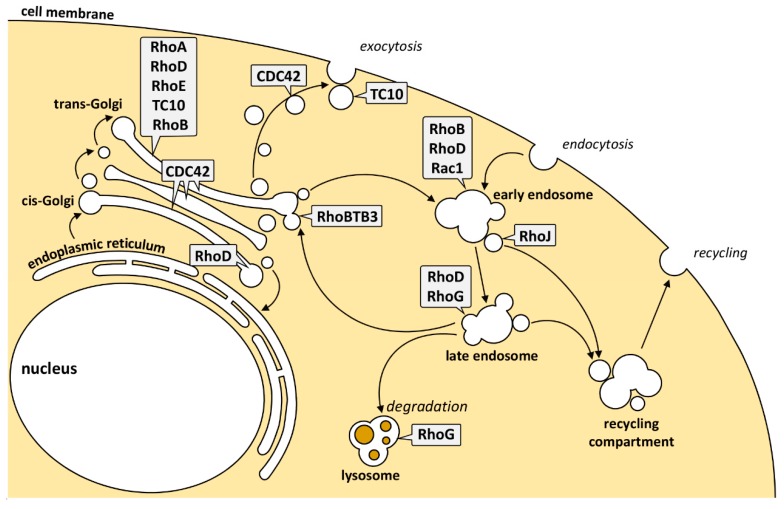
Rho GTPAses in intracellular trafficking pathways. For a detailed description see text.

Collectively, these data show that several pools of individual Rho GTPases are present at membranes of the endocytic and exocytic pathway (Figure 2). The signaling pathways emanating from these local Rho pools are diverse and dictated by the presence of the GEFs, GAPs, and effector proteins at these sites (Table 1).

## 6. Rho GTPases and Membrane Trafficking—Implications for Cancer

As opposed to Ras GTPases, which are hyperactivated in approximately a quarter of all human cancers [144], dysregulation of Rho GTPase signaling has mainly been attributed to Rho GTPase overexpression or altered GEF or GAP levels. Especially GEF proteins such as Ect2 (ARGHEF31), Prex1, Vav1, Tiam1, or GEF-H1 have been connected with the growth and progression of various cancers [145]. Only recently, mutations in Rho GTPases have been identified and causally linked with specific subsets of cancers. For example, recurrent hot-spot mutations in RhoA have been described in peripheral T-cell lymphomas [146,147,148,149] and diffuse gastric carcinomas [150,151]. In the case of Rac, a Rac1b splice variant was initially found in colon and breast cancers, and, more recently, a Rac1/P29S mutant has been associated with melanoma, whereas somatic mutations in Cdc42 have been described in papillary mesothelioma [152]. However, the precise downstream pathways accounting for the transforming roles of Rho GTPases in these cancers are largely unexplored, and whether aberrant membrane trafficking contributes to the transforming potential of these mutant Rho GTPases is unknown. Intriguingly, the transforming activity of constitutively active Cdc42F28L was shown to depend on its interaction with the gamma subunit of the coatomer complex, the major coat component of Golgi-derived COPI transport vesicles, and ectopic expression of Cdc42F28L resulted in endosomal EGFR accumulation and prolonged EGFR signaling [19,141]. This indicates that altered membrane trafficking is associated with the tumor-promoting functions of Cdc42. Altered endocytic trafficking was further shown to be crucial for the oncogenicity of RTKs such as c-Met [153,154]. Constitutively active, oncogenic Met mutants accumulated in endosomal compartments and were characterized by enhanced endosomal Rac1 activity, reduced actin stress fibers, and increased cell migration. This highlights the important contribution of dysregulated endocytic signaling to cell transformation and suggests that endosomal Rac1 signaling contributes to tumor progression triggered by oncogenic c-Met mutants. Regardless of the precise mechanisms by which Rho GTPases dysregulation occurs in cancer, it is clear that the high proliferation and migration rates of cancer cells rely on robust and active membrane trafficking machinery. The polarized delivery of proteins to apical and basolateral membranes in epithelial cells and membrane flow as a driving force of invasive cell motility provide further obvious connections between aberrant Rho GTPase signaling, neoplastic transformation, and tumor progression [155].

Early evidence for altered membrane trafficking promoting the tumor phenotype has been provided for RhoB. Whereas farnesylated RhoB was shown to possess either pro- or anti-proliferative activity depending on the precise context, the geranylgeranylated, endomembrane-associated RhoB pool has been implicated in growth inhibition. Specifically, when farnesylation was blocked by farnesyltransferase inhibitors (FTIs), which were originally developed to inhibit the prenylation and oncogenic activity of Ras, geranylgeranylated RhoB species accumulated in the cells and RhoB was found to be responsible for the growth inhibitory effect of FTIs. Indeed, studies with RhoB nullizygous cells, which were resistant to FTI-induced apoptosis both in vitro and in vivo, provided genetic proof for RhoB being a crucial mediator of the antineoplastic effects of FTIs [156,157].

These location-dependent differences in RhoB function provide an explanation for the contradictory results on RhoB functioning as an oncogene or tumor suppressor. For example, inhibition of RhoB was shown to promote migration and invasion of bronchial cells via an Akt kinase-dependent mechanism, most likely via a pathway later shown to involve inactivation of the phosphatase PP2A [158,159]. However, there is also evidence for RhoB positively cooperating with Akt signaling downstream of activation. For example, RhoB protected human keratinocytes from UVB-induced apoptosis through EGFR signaling [160]. Similarly, in RhoB-null vascular smooth muscle cells, PDGF receptor trafficking to the late endosomal compartment and Akt activation were compromised, and this was later shown to also impair Cdc42 and Rac transport to the PM [124,161]. Intriguingly, a recent study also showed that high expression levels of the small GTPase RhoB in non-small-cell lung cancer (NSCLC)conferred resistance to EGFR-TKIs by enhancing Akt activation [162], although it was not investigated whether this was connected to altered EGFR trafficking. Since Akt signaling emanates from both the PM and endomembranes and RhoB is also involved in the nuclear translocation of Akt [163], the precise function of RhoB depends on its location, the available effector proteins and the nature of activated downstream signaling pathways in the cells. This is exemplified in a very recent paper that identifies Arf6 as a factor crucial for RhoB localization to endosomes in addition to the *CAAX* motif. Notably, Arf6 depletion impaired endosomal localization of RhoB followed by its degradation through an endolysosomal pathway. Most importantly, Arf6 or RhoB depletion enhanced the c-Met-dependent 3D migration of invasive breast cancer cells [164]. In sum, RhoB appears to play a general role in the trafficking decisions of diverse membrane-associated proteins, including RTKs, adhesion receptors, and signaling molecules, such as Src and GTPase family members, which together influence the cancer cell phenotype.

The members of the DLC family have been established as tumor suppressors, with DLC3 shown to localize at RhoB and Rab8 positive endomembranes, in addition to sites of cell-cell and cell-matrix adhesions [39]. DLC3 was reported to be downregulated in various types of cancers, including breast, prostate, kidney, lung, and ovarian [165]. DLC3 knockdown prevented EGFR degradation and enhanced Akt activation by trapping the receptor in EEA1-positive endosomes [41], making it tempting to speculate that reduced DLC3 expression might also confer EGFR-TKI resistance as described for lung cancers overexpressing RhoB [162]. DLC3 depletion in breast cancer cells also caused aberrant Rab4-dependent matrix metalloproteinase recycling and invadopodia matrix degradation in a RhoB- and actin-dependent manner [41,42,166]. These findings clearly manifest a role for DLC3 in the coordination of endocytic membrane trafficking steps, which most likely contribute to DLC3 requirements for the formation of stable adherens junctions and lumenogenesis in polarized epithelial cells.

Enhanced activation of Cdc42 has been shown to modify the ability of oncoproteins, including Ras and EGFR, to induce cellular transformation [167]. In the case of oncogenic H-Ras, the cellular pools at the PM and endomembrane have been shown to elicit distinct downstream signaling. Interestingly, signaling at the PM, which was sufficient to activate Raf1, was only weakly transforming, whereas activation of Cdc42 by Ras at the endomembrane was required to achieve full transformation [168]. A more recent study postulated that Cdc42 activated at Golgi membranes is transported to the leading edge of cells to sustain asymmetric front-rear Cdc42-GTP distribution during directed migration [33]. Dysregulation of the mechanisms that control Cdc42 activity at Golgi membranes is, thus expected to favor cancer cell motility. Several GEFs with potential oncogenic activity are implicated in Cdc42 activation at the Golgi membranes, including Tuba, Dbs, FGD1, and FGD4 (described in detail above and [169]). Inhibition of the Dbs-130 isoform, in particular, was shown to impair the reorientation of the Golgi toward the leading edge, and consequently, cell motility [71]. Similarly, FGD1 was shown to drive invadopodia biogenesis and extracellular matrix degradation in an invasive cell model by modulating Cdc42 activation [170]. The transforming activities of Dbs-130 and FGD1 might, thus involve their roles in secretory pathway regulation. Whether Golgi-localized GAP proteins such as ARHGAP21, with decreased expression in ovarian cancer [171] or DLC3, might counteract Cdc42 activation at Golgi membranes in this respect has not been addressed. Last but not least, RhoA and Cdc42 have been implicated in the matrix-degrading function of invadopodia by triggering the engagement of the Rho effector protein IQGAP with components of the exocyst complex involved in MT1-MMP trafficking to invadopodia [172]. It thus appears plausible that the cytoskeleton remodeling function of Rho GTPases is coordinated with the exocytic vesicle-tethering machinery for the generation of protrusions and for cancer cell invasiveness.

## 7. Conclusions and Outlook

Our knowledge of the regulation of membrane trafficking through Rho GTPases has vastly increased in the past years. The development of biosensors for live cell imaging with high specificity and sensitivity has pushed the field ahead and confirmed the existence of local Rho GTPase pools acting on endomembranes of the endocytic and exocytic pathways. The identification of Rho effectors at these sites has further extended the functions attributed to Rho GTPases beyond cytoskeletal remodeling. However, a remaining challenge is the identification of the RhoGEF and RhoGAP proteins that keep local Rho activity in check. Although large biochemical interaction screens have provided valuable detailed information on the interaction network of Rho GTPases, they are limited by the lack of spatial information. Here, proximity labeling techniques such as BioID or APEX hold promise for identifying protein-protein interactions with spatial and temporal resolution and have already been successfully applied to identify novel interactors of Rho GTPases [173].

The cellular function of Rho GTPases and their GEFs and GAPs has been extensively studied by protein depletion through gene knockout or knockdown. Although these approaches provide useful information about gene function, they also harbor some drawbacks. The generation of knockout cell lines requires the cultivation of cells for an extended period of time, and the constitutive gene knockout can activate compensatory mechanisms that may mask the phenotype. In contrast, gene knockdown through RNAi is achieved much faster but fully depends on the half-life of the protein, which might be in the range of hours and days. Here, the application of next-generation techniques such as the auxin-inducible degradation technology or proteolysis-targeting chimeras (PROTACs) would allow for rapid and controlled protein depletion to detect primary molecular responses while avoiding secondary, indirect effects of protein dysregulation [174,175].

Yet another experimental challenge is provided by the fact that RhoGEFs and RhoGAPs dynamically change their intracellular location and reside only transiently on target membranes to regulate Rho activity. An additional layer of complexity is added because Rho GTPases are trafficked themselves. This underscores the need for new methods, specifically allowing for the spatiotemporal investigation of subcellular Rho pools. An excellent approach is the use of optogenetics to reversibly trigger signaling with spatial and temporal control in the subsecond range [176,177]. Such light-sensitive tools have already been successfully developed to control the activity of endogenous Rho GTPases in various settings [178,179,180] and can easily be combined with genetically encoded biosensors for monitoring local Rho GTPase or RhoGEF activity [47,62,64].

## Figures and Tables

**Table 1:** Rho GTPases, GEFs, GAPs, and effector proteins on endocytic and exocytic membranes.

Rho GTPase	Location	GEF	GAP	Effectors	Function	Reference
RhoA	TGN	GEF-H1 Trio (?)	DLC3	PLCε ROCK	Promotes cargo transport to the PM; Formation of Golgi outposts in neuronal cells	[41,46,82,83,84,85,87,89]
RhoB	Endosomes	Vav2 (?)	DLC3	mDia1 mDia2 WAVE1	Transport from endosomes to the lysosomes; Src movement and activation	[31,41,42,110,111,113,114,115]
	Golgi complex	?	?	?	?	[46,81]
Cdc42	Golgi complex; Exocytic vesicles	Intersectin-1, Tuba, FGD1, Dbs-130	ARHGAP21	ARP2/3, N-WASP; FMNL2/3	Anterograde trafficking through the Golgi; Golgi-to-PM transport; Retrograde trafficking from the Golgi to the ER	[33,53,54,55,56,57,58,59,60,61,62,65,66,68,70,71,72,76,78,81,140]
	Early endosomes	β-Pix	?	?	Ccd42 delivery to the leading edge during directed cell migration	[55,141]
RhoD	Trans-Golgi membranes	-	-	WHAMM	Golgi-to-PM transport	[95,96]
	Endosomes	-	-	hDia2C	Inhibition of endosomal motility; Regulation of RTK trafficking	[119,120,121,122,142]
RhoE	Trans-Golgi membranes	-	-	ROCK1	Inhibits RhoA signaling through competing in binding to ROCK1	[93,94]
RhoBTB3	Golgi complex; Vesicular structures	-	-	Cyclin E	Promotes retrograde trafficking from the late endosomes to the Golgi; Required for S-phase progression	[91,92,137]
TC10	Golgi complex; Exocytic vesicles	ARHGEF10 (?)	p190RhoGAP	PIST Exo70	Cargo loading at the TGN; Promotes exocytic vesicle fusion with the PM	[81,98,100,101,102,103,143]
Rac1	Endosomes	TIAM1 Vav2	?	?	Important for Rac1 activation and delivery to the leading edge; Formation of actin-based migratory protrusions; Promotion of cell migration and invasion	[124,127]
RhoJ/TCL	Early endosomes	?	?	?	TfR recycling	[134]
RhoG	Lysosomes	?	?	?	Lysosomal dynamics	[135]
